# Multimodal-based machine learning strategy for accurate and non-invasive prediction of intramedullary glioma grade and mutation status of molecular markers: a retrospective study

**DOI:** 10.1186/s12916-023-02898-4

**Published:** 2023-05-29

**Authors:** Chao Ma, Liyang Wang, Dengpan Song, Chuntian Gao, Linkai Jing, Yang Lu, Dongkang Liu, Weitao Man, Kaiyuan Yang, Zhe Meng, Huifang Zhang, Ping Xue, Yupeng Zhang, Fuyou Guo, Guihuai Wang

**Affiliations:** 1 School of Clinical Medicine, Tsinghua University, Beijing, China; 2 Department of Neurosurgery, Beijing Tsinghua Changgung Hospital, School of Clinical Medicine, Tsinghua University, Beijing, China; 3 Hepato-Pancreato-Biliary Center, Beijing Tsinghua Changgung Hospital, School of Clinical Medicine, Tsinghua University, Beijing, China; 4 Department of Neurosurgery, The First Affiliated Hospital of Zhengzhou University, Zhengzhou University, Zhengzhou, China; 5 Institute for Precision Medicine, Tsinghua University, Beijing, China; 6 State Key Laboratory of Low-Dimensional Quantum Physics and Department of Physics, Tsinghua University, Collaborative Innovation Center of Quantum Matter and Beijing Advanced Innovation Center for Structural Biology, Beijing, 100084 China; 7 Department of Neurosurgery, Beijing Tiantan Hospital, Capital Medical University, Beijing, 100070 China

**Keywords:** Intramedullary gliomas, Alpha thalassemia/mental retardation syndrome X-linked, Tumor protein p53, Multimodal, Machine learning

## Abstract

**Background:**

Determining the grade and molecular marker status of intramedullary gliomas is important for assessing treatment outcomes and prognosis. Invasive biopsy for pathology usually carries a high risk of tissue damage, especially to the spinal cord, and there are currently no non-invasive strategies to identify the pathological type of intramedullary gliomas. Therefore, this study aimed to develop a non-invasive machine learning model to assist doctors in identifying the intramedullary glioma grade and mutation status of molecular markers.

**Methods:**

A total of 461 patients from two institutions were included, and their sagittal (SAG) and transverse (TRA) T2-weighted magnetic resonance imaging scans and clinical data were acquired preoperatively. We employed a transformer-based deep learning model to automatically segment lesions in the SAG and TRA phases and extract their radiomics features. Different feature representations were fed into the proposed neural networks and compared with those of other mainstream models.

**Results:**

The dice similarity coefficients of the Swin transformer in the SAG and TRA phases were 0.8697 and 0.8738, respectively. The results demonstrated that the best performance was obtained in our proposed neural networks based on multimodal fusion (SAG-TRA-clinical) features. In the external validation cohort, the areas under the receiver operating characteristic curve for graded (WHO I–II or WHO III–IV), alpha thalassemia/mental retardation syndrome X-linked (*ATRX*) status, and tumor protein p53 (*P53*) status prediction tasks were 0.8431, 0.7622, and 0.7954, respectively.

**Conclusions:**

This study reports a novel machine learning strategy that, for the first time, is based on multimodal features to predict the *ATRX* and *P53* mutation status and grades of intramedullary gliomas. The generalized application of these models could non-invasively provide more tumor-specific pathological information for determining the treatment and prognosis of intramedullary gliomas.

**Supplementary Information:**

The online version contains supplementary material available at 10.1186/s12916-023-02898-4

## Background

Intramedullary gliomas (IMGs) are the most common primary spinal cord neoplasms, accounting for approximately 80% of spinal cord tumors and 2–4% of central nervous system tumors [1]. The tumor grade and genetic and histological characteristics of gliomas are considered to affect their prognosis and response to treatment. The fifth edition of the WHO Classification of Tumors of the Central Nervous System (WHO CNS5) promotes the application of molecular characteristics for diagnosis and grading. In general, pathological examination and immunohistochemistry (IHC) during surgery or biopsy are required to analyze tumor molecular biomarkers [2, 3]. Without pathological guidance, therapeutic options for patients who are unsuitable for surgery or those who choose nonsurgical therapy may be limited. Although relatively safe, these invasive examinations can damage the normal brain or spinal cord tissue. Owing to the highly dense neural structure of the spinal cord, any minor injury may cause permanent damage to bodily function [4]. Hence, biopsy is not suitable for IMGs, and thus, the demand for alternative non-invasive approaches that can offer genetic and histological evidence of IMGs has surged [5].

Accurate classification of IMG preoperatively is crucial for physicians to develop an appropriate treatment plan. Preoperative magnetic resonance imaging (MRI) remains the most widely utilized and efficient technique for detecting spinal cord lesions in clinical practice. Increasing evidence has revealed the feasibility of using MRI to predict the type of glioma and molecular biomarkers, such as IDH1, Ki67, and H3-K27M, via machine learning or deep learning method [6–8]. However, only a limited number of studies have attempted to predict markers and classification of IMGs based on MRI owing to the lack of sufficient training in such rare tumors [9, 10].

Although IMGs and brain gliomas are both derived from glial cells, the molecular characteristics of primary IMGs are significantly different from those of brain gliomas [11, 12]. As summarized in the WHO CNS5, the mutation-site genotypes of genes such as isocitrate dehydrogenase (*IDH*), tumor protein p53 (*P53*), and alpha thalassemia/mental retardation syndrome X-linked (*ATRX*) are important indicators for the classification of gliomas [13]. Despite the commonly mentioned *IDH* mutations in brain gliomas, *IDH* mutations are rare in IMGs. The absence of *IDH* mutations in the spine cannot be used to distinguish between grade I and II diffuse astrocytomas. Moreover, IDH IHC would be ineffective because most IMGs lack the conventional *IDH1* p.R132H mutation [12]. Hence, determining the mutation status of *ATRX* and *P53* in IMGs is especially important.

## Methods

### Aim

The present study aimed to develop a non-invasive preoperative method for predicting IMG grade and mutational status of molecular markers. Towards this goal, we retrospectively analyzed the preoperative MRI scans of patients with IMGs whose *ATRX* and *P53* mutation statuses were tested by IHC. In the experimental design, multiple feature representations and mainstream machine learning models were compared to explore the superior predictive effect. After exploring the superior feature representation of novel machine learning models, including WHO-Mind, ATRX-Mind, and P53-Mind, methods were developed to accurately classify the glioma grade and predict the *ATRX* and *P53* mutation statuses of IMGs. Additionally, rigorous external validation was performed to verify the generalizability of the proposed method.

### Patient demographic characteristics and data acquisition

This retrospective study was approved by the ethical committees of Beijing Tsinghua Changgung Hospital (hospital 1) and The First Affiliated Hospital of Zhengzhou University (hospital 2) and was conducted in accordance with the principles of the Declaration of Helsinki. The requirement for obtaining informed consent was waived.

We collected the records of patients from two hospitals (hospital 1: January 2015 to April 2021 and hospital 2: February 2017 to October 2021). The patient inclusion criteria were preoperative MRI examination, pathological diagnosis of IMGs according to the WHO CNS5, and IHC-confirmed *ATRX* and *P53* mutation status. Patients who had undergone biopsy, chemotherapy, and radiotherapy and those with IMG metastases from brain glioma were excluded. Following the initial patient screening, MRI data were evaluated, and the exclusion criteria were as follows: absence of sagittal (SAG) and transverse (TRA) T2-weighted imaging (T2WI) scans and artifacts in the MRI image.

Data on clinical baseline characteristics, including age, sex, smoking, alcohol consumption, medical history with accompanying diseases, time of onset, preoperative McCormick score, and radiological features of tumor-related lesions, were collected. Moreover, all radiological features, including tumor number, axis ratio (tumor/spinal cord), bleeding, cysts, spinal cord cavity, edema, atrophy, and malformation, were independently assessed by three neuroradiologists. All of these features were not obtainable by radiomic feature extraction. Two experienced pathologists performed the pathological evaluation of all gliomas using the WHO CNS5 for glioma grading and classification [13]. We classified glioma grades as low grade (WHO I–II, benign) and high grade (WHO III–IV, malignant), and the mutation status of *ATRX* and *P53* was determined by IHC.

**Fig. 1:**
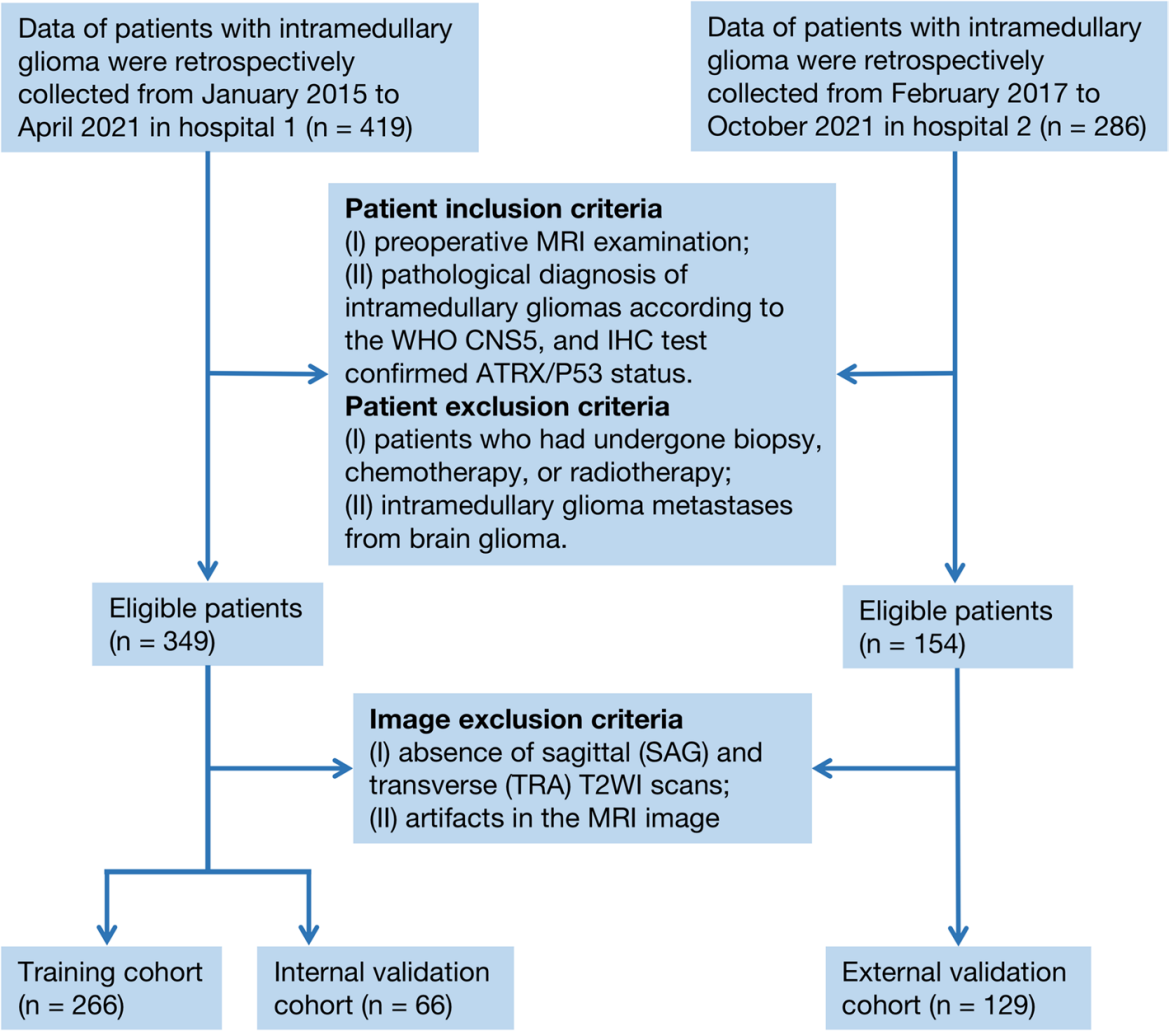
Flow diagram showing the patient selection protocol and inclusion and exclusion criteria. ATRX, alpha thalassemia/mental retardation syndrome X-linked; IHC, immunohistochemistry; P53, tumor protein p53; SAG, sagittal; TRA, transverse; WI, weighted imaging

Patients recruited from hospital 1 were included in the primary cohort (*n* = 332) and were randomly assigned to the training set and internal validation set after fivefold cross-validation according to the *ATRX*/*P53* mutation status and tumor grade. Patients from hospital 2 were included in the independent external validation cohort (*n* = 129). A detailed flow diagram of the patient selection process is illustrated in Fig. 1.

### MRI acquisition and region of interest segmentation

MR data were obtained employing a 3.0-T MR scanner (Discovery MR750; GE Healthcare, Milwaukee, WI) in hospital 1 and either Siemens (Verio, Prisma, or Skyra; Siemens Healthcare, Erlangen, Germany) or Philips (Ingenia, Philips Medical System, Best, The Netherlands) MR systems in hospital 2. An overview of the parameters used to acquire MRI is given in Additional file 1, which shows that scans are obtained by scanners from different vendors employing various acquisition settings. Identifying the borders of low-grade gliomas is difficult on T1-weighted and contrast-enhanced sequences; thus, the T2-weighted (T2W) sequence is well accepted for identifying abnormal hyperintense signals representing gliomas, and tumor regions of interest (ROIs) were delineated on SAG and TRA T2WI separately [14].

Lesions were delineated on both SAG and TRA T2WI by two experienced neuroradiologists using the ITK-SNAP software (www.itksnap.org). Abnormal hyperintense signals on T2W MR images were regarded as tumor regions, and the signals of cerebrospinal fluid, spinal cord cavity, and edema were avoided. If the ROI was controversial, a senior neuroradiologist performed the final determination. T1-weighted images with and without gadolinium enhancement, short T1 inversion recovery, and fluid attenuation inversion recovery images were collected for most patients to aid in identifying tumors. Only T2WI was used in subsequent studies. Finally, to reduce the heterogeneity between different scanners, we resampled the T2W images and the corresponding ROI mask into a uniform voxel size of 0.5 × 0.5 × 5 mm across all patients for radiomic extraction.

### Lesion segmentation using deep learning

After preprocessing, deep learning models were proposed to segment lesions, which greatly improved future work efficiency. This work selected a deep learning model method based on the transformer architecture (Swin transformer) because of its superiority in multiple domains [15, 16]. The Swin transformer adopts a hierarchical design containing a total of four stages: each stage decreases the resolution of the input feature map and expands the perceptual field layer by layer, similar to a convolutional neural network. In addition, it is designed with patch embedding, which cuts MRI scans into patches and embeds them into the model. Although this architecture has performed well in several tasks [17, 18], its use for segmenting IMGs has not been reported.

Both SAG and TRA images of the primary cohort were employed for the training, internal, and external validation cohorts for testing. The input channel was two dimensional, that is, all MRI sequences were converted into slices and then batch inputted, and the final output results were reconstructed in 3D. Transfer learning was adopted for training, and the pre-trained model was swin_transformer_base_patch4_window7_224_imagenet_1k. The backbone of the model was SwinTransformer_base_patch4_window7_224.

### Feature extraction and selection

Radiomic features were extracted using the PyRadiomics module written in Python 3.7 in accordance with a previous study [19]. Both the SAG and TRA images extracted the following six feature classes: first-order statistics, shape, gray-level run-length matrix, gray-level size zone matrix, neighboring gray-tone difference matrix, and the gray-level dependence matrix. These feature classes used six built-in filters (wavelet, square, square root, logarithm, exponential, and gradient filters). All features were named by connecting the MRI type, the image type from which the feature was extracted, the feature class, and the feature name separated by an underscore. For example, TRA_original_glrlm_RunEntropy is a feature extracted from the TRA T2WI sequence, original image, and glrlm feature class, and the feature name is RunEntropy.

For every patient, 1960 radiomic features were extracted from SAG and TRA T2WI data, and all radiomic features were *z* transformed for data standardization. To avoid interobserver variations during manual segmentation, we calculated the intraclass correlation coefficient (ICC) for each feature, and only those with high stability (ICC > 0.8) were included in the analysis [20]. Then, the selected stable features were tested using the independent samples *t*-test or the Mann–Whitney *U* test to select potential important features. Features that did not meet the criteria for either of the aforementioned tests were excluded. This study adopted the least absolute shrinkage and selection operator (LASSO) on the training cohort to screen significant features with non-zero coefficients that can differentiate *ATRX* and *P53* mutation status or glioma grade separately. For the three outcomes of ATRX, P53, and tumor grade, we used LASSO to select features in the TRA, SAG, and TRA + SAG groups, respectively. The aforementioned calculation methods are available in PyRadiomics 2.2.0 documentation [21].

### Prediction model construction

In this study, three types of deep neural networks (DNNs) were constructed based on the data characteristics of the prediction tasks: WHO-Mind, ATRX-Mind, and P53-Mind. WHO-Mind has one input layer, four hidden layers, and one output layer. Both ATRX-Mind and P53-Mind have one input layer, three hidden layers, and one output layer. For each DNN, the ReLU and Adam were selected as the activation function and the solver for weight optimization, respectively. In addition, the initial learning rates and batch sizes were set to 0.01 and 64 in all models, respectively. The model architecture and the key hyperparameters are summarized in Additional file 2. However, the unbalanced nature of the data included in this study posed a challenge for model training, as data category imbalance may lead to severe overfitting. Therefore, after dividing the dataset, the Synthetic Minority Oversampling Technique (SMOTE) was adopted for the divided training set to achieve data augmentation. Its basic idea is to generate new synthetic samples by interpolating between minority class samples, thereby balancing the class distribution, and thus improving the performance of the classifier.

Specifically, this algorithm analyzes the minority class samples and artificially synthesizes new samples based on the minority class samples to add to the dataset, thereby solving the problem of model overfitting. The algorithm first randomly selects a sample from the minority class (sample A) and identifies its *k* nearest neighbors within the minority class. Subsequently, a neighbor (sample B) is randomly chosen from these *k* neighbors. The new synthetic sample is created on the line segment between sample A and sample B. This process is repeated as needed until a sufficient number of synthetic minority class samples are generated to achieve the desired class balance. It is worth noting that SMOTE solely creates new samples within the feature space without generating new class labels. Synthetic samples inherit the class label of their parent samples (i.e., sample A and sample B). To implement SMOTE in Python 3.7, we utilized the imbalanced-learn library, applying the SMOTE algorithm to the training data in order to balance the class distribution.

Additionally, considering that some mainstream models that have been developed obtained good performance in related fields [22–24], extreme Gradient Boosting (XGBoost), Gradient Boosting Decision Tree (LightGBM), and random forest (RF) were also trained and tested for comparison. The parameters of the above models were optimized during the training process using the GridSearchCV tool in Scikit-learn 1.1.1. The modeling was performed in the Python 3.7 programming environment, and the core library involved was Scikit-learn 1.1.1.

### Experimental setup for the prediction models

**Fig. 2:**
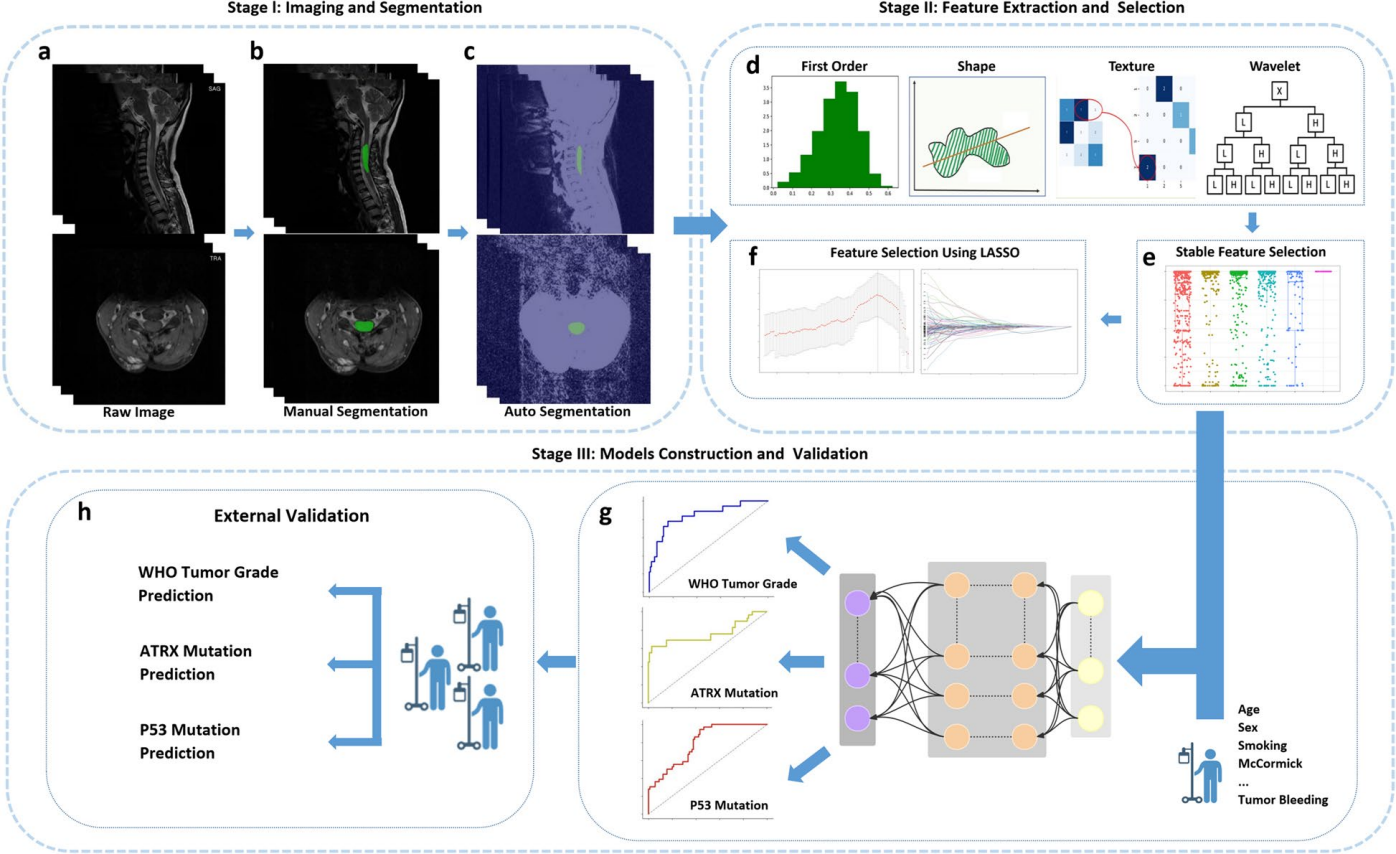
Illustration of the study process. Stage I includes raw image acquisition (**a**), manual ROI segmentation (**b**), and auto ROI segmentation (**c**). Stage II includes feature extraction and selection. From both SAG and TRA images, radiomic features, including first-order statistical, shape, texture, and wavelet features, are extracted (**d**). All extracted features are screened out by ICC to select stable features (**e**). Informative features are then selected using LASSO (**f**). Stage III includes model construction and validation. Selected clinical and radiomic features are entered into the deep neural networks to predict the different tasks (**g**), and model performance is further tested in the external validation cohort (**h**). ATRX, alpha thalassemia/mental retardation syndrome X-linked; ICC, intraclass correlation coefficient; LASSO, least absolute shrinkage and selection operator; P53, tumor protein p53; ROI, region of interest; SAG, sagittal; TRA, transverse

The overall process of this study is illustrated in Fig. 2. Three tasks (WHO tumor grade, *ATRX* mutation status prediction, and *P53* mutation status prediction) were included, and each was used to build four machine learning models. Moreover, six different feature representations were compared to explore the most superior model input: SAG radiomics, SAG radiomics with clinical baseline, TRA radiomics, TRA radiomics with clinical baseline, SAG + TRA radiomics, and SAG + TRA radiomics with clinical baseline features. In the primary cohort, fivefold cross-validation was employed to identify the best models. Data were randomly divided into five equal parts: one was selected for internal validation, and the rest were trained. This process was repeated five times. The best-performing models were tested using an external validation cohort to verify the generalization ability. This work was completed in Windows 10 OS and involved computing devices with a CPU AMD Ryzen 7 5800H (16 GB RAM) and GPU GeForce RTX ™ 3090 (24 GB RAM).

### Statistical analysis

When representing continuous variables, differences were assessed using Student’s *t*-test or the Mann–Whitney *U* test, as appropriate, and data are represented as median with interquartile range. Specifically, because the fivefold cross-validation was employed in the primary cohort, we also calculated the means and 95% confidence intervals (CIs) for the evaluation metrics of the models. We adopted the chi-square test to evaluate the differences in categorical variables, and the results are presented as the number of events and relative frequency (%). The performance of all models was evaluated according to accuracy (Acc), sensitivity (Sens), specificity (Spec), *F*_1_ scores, and receiver operating characteristic (ROC) curves. A dice similarity coefficient (DSC) was adopted to evaluate the performance of the networks for IMG segmentation. All statistical analyses were performed employing R software (version 3.6.3, R Foundation for Statistical Computing, Vienna, Austria). Statistical significance was defined as *p* ≤ 0.05.

## Results

### Clinicodemographic patient characteristics

A total of 332 patients in hospital 1 (primary cohort) were divided into training and internal validation cohorts, and 127 patients in hospital 2 were enrolled as the independent external validation cohort. The clinical baseline features of the patients and the distribution of *ATRX* and *P53* mutation status across the primary and external validation cohorts are presented in Additional file 3. In the primary cohort, 52 patients (15.7%) had high-grade gliomas and 280 patients (84.3%) had low-grade gliomas. The external validation cohort included 20 (58.8%) patients with high-grade gliomas and 14 (41.2%) patients with low-grade gliomas. There were significant differences in the numbers of *ATRX* (79/332, 23.8%; 43/127, 33.9%; *p* = 0.039) and *P53* (72/332, 21.7%; 44/127, 34.6%; *p* = 0.006) mutations between the primary and external validation cohorts. Meanwhile, no significant difference was found in age, sex, onset month, accompanying diseases, smoking, alcohol consumption, or the McCormick score between the primary and external validation cohorts. Regarding the imaging features extracted by neuroradiologists, the axis ratio of the tumor in the spinal cord and the proportion of tumor bleeding differed significantly between the two hospitals.

### Results of lesion segmentation

The Swin transformer model was trained using slices of SAG and TRA images and artificially labeled lesions in the primary cohort. After 50,000 iterations, the model proved to be completely convergent and had no overfitting. Therefore, this model was selected for testing at this point. In total, 20% of the samples were randomly selected from patients as the test set, and the rest were used as the training set. In the SAG phase test, the Acc and DSC of the automatic segmentation model reached 0.9929 and 0.8697, respectively. In the TRA phase test, the Acc and DSC of the automatic segmentation model were 0.9978 and 0.8738, respectively. These results demonstrated that the aforementioned deep learning model was relatively satisfactory for the segmentation of lesions in this study. Visualization of the automatic segmentation of the two phases is demonstrated in Fig. 2c.

### Feature extraction and selection

For every patient, 1960 radiomic features were derived, and the ICC results showed that 1572 (80.1%) features were stable. The selected stable features in the TRA and SAG groups are listed in Additional file 4. A total of 1560, 1554, and 1517 features for the *ATRX* mutation status, *P53* mutation status, and WHO tumor grading tasks, respectively, passed the scrutiny of the *t*-test or the Mann–Whitney *U* test. We employed fivefold cross-validation in LASSO for each prediction task to select the meaningful features. In the WHO tumor grade prediction task, 24 features were selected in SAG, 6 features in TRA, and 20 features in SAG + TRA (Additional file 5). In the *ATRX* mutation status prediction task, 3 features were selected in SAG, 39 features in TRA, and 5 features in SAG + TRA (Additional file 6). In the *P53* mutation status prediction task, 21 features were selected in SAG, 24 features in TRA, and 57 features in SAG + TRA (Additional file 7). All features selected by LASSO are listed in Additional file 8.

### Results of prediction models in the primary cohort

**Fig. 3:**
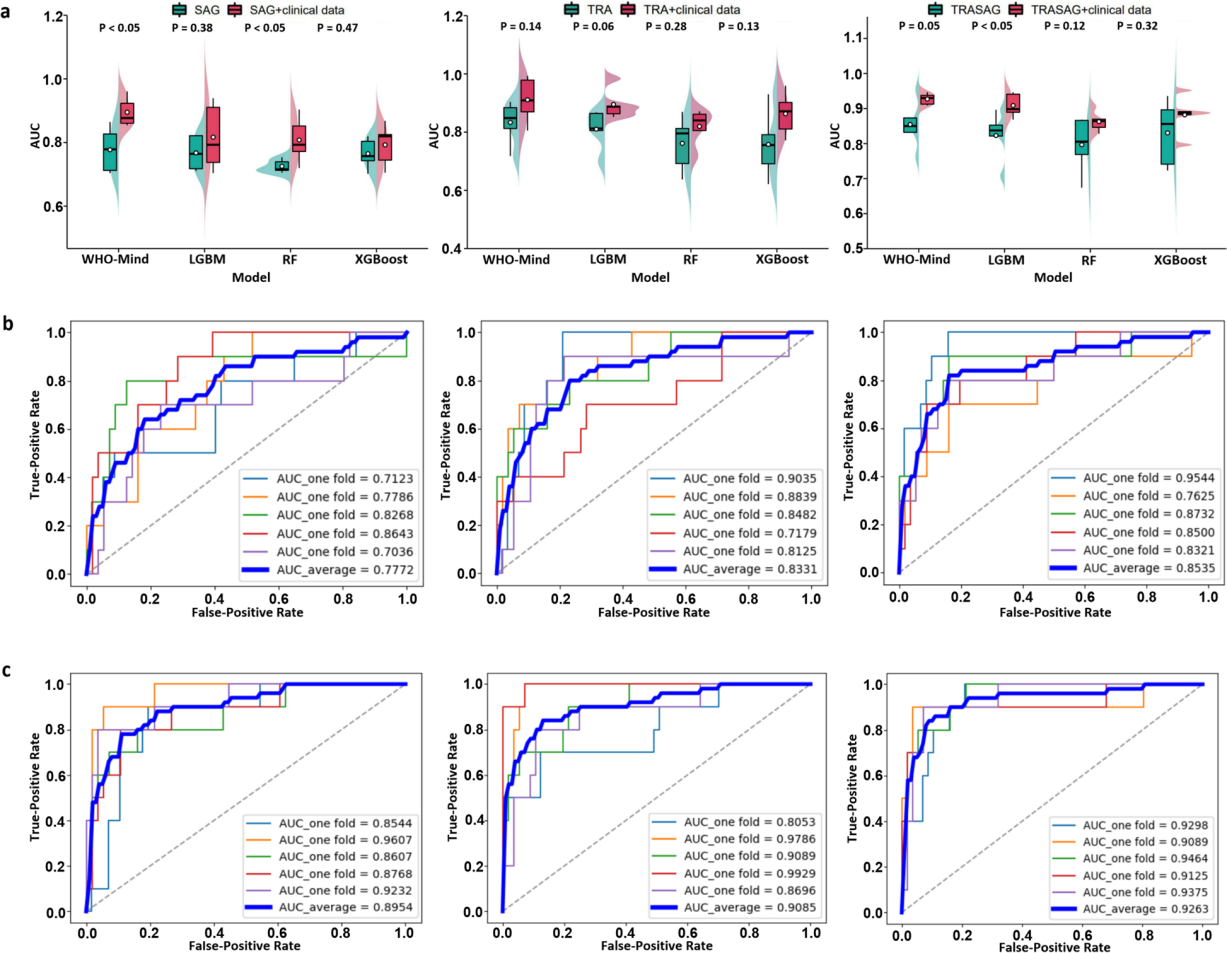
The fivefold cross-validation results in the WHO prediction task. **a** The comparison results of the proposed WHO-Mind with the mainstream machine learning models. **b** The receiver operating characteristic (ROC) curves of WHO-Mind when inputting features of the SAG phase (left), TRA phase (middle), and SAG combined with TRA phase (right). **c** The ROC curves of WHO-Mind when the features of the SAG phase-clinical baseline (left), TRA phase-clinical baseline (middle), and SAG phase-TRA phase-clinical baseline (right) are inputted. AUC, area under the curve; SAG, sagittal; TRA, transverse

**Fig. 4:**
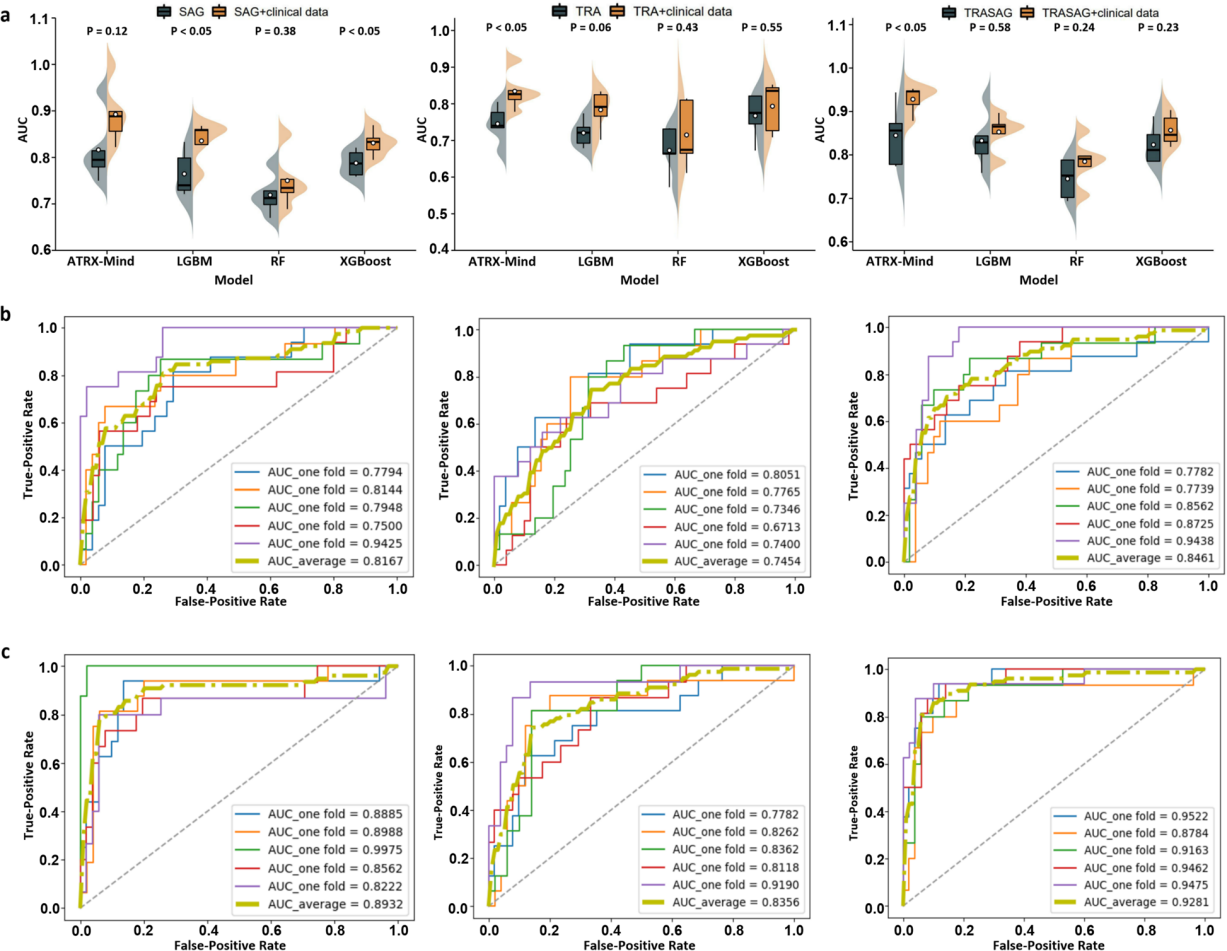
The fivefold cross-validation results in the *ATRX* genotype prediction task. **a** The comparison results of the proposed ATRX-Mind with the mainstream machine learning models. **b** The ROC curves of ATRX-Mind when inputting features of the SAG phase (left), TRA phase (middle), and SAG combined with TRA phase (right). **c** The ROC curves of ATRX-Mind when the features of the SAG phase-clinical baseline (left), TRA phase-clinical baseline (middle), and SAG phase-TRA phase-clinical baseline (right) are inputted. ATRX, alpha thalassemia/mental retardation syndrome X-linked; AUC, area under the curve; ROC, receiver operating characteristic; SAG, sagittal; TRA, transverse

**Fig. 5:**
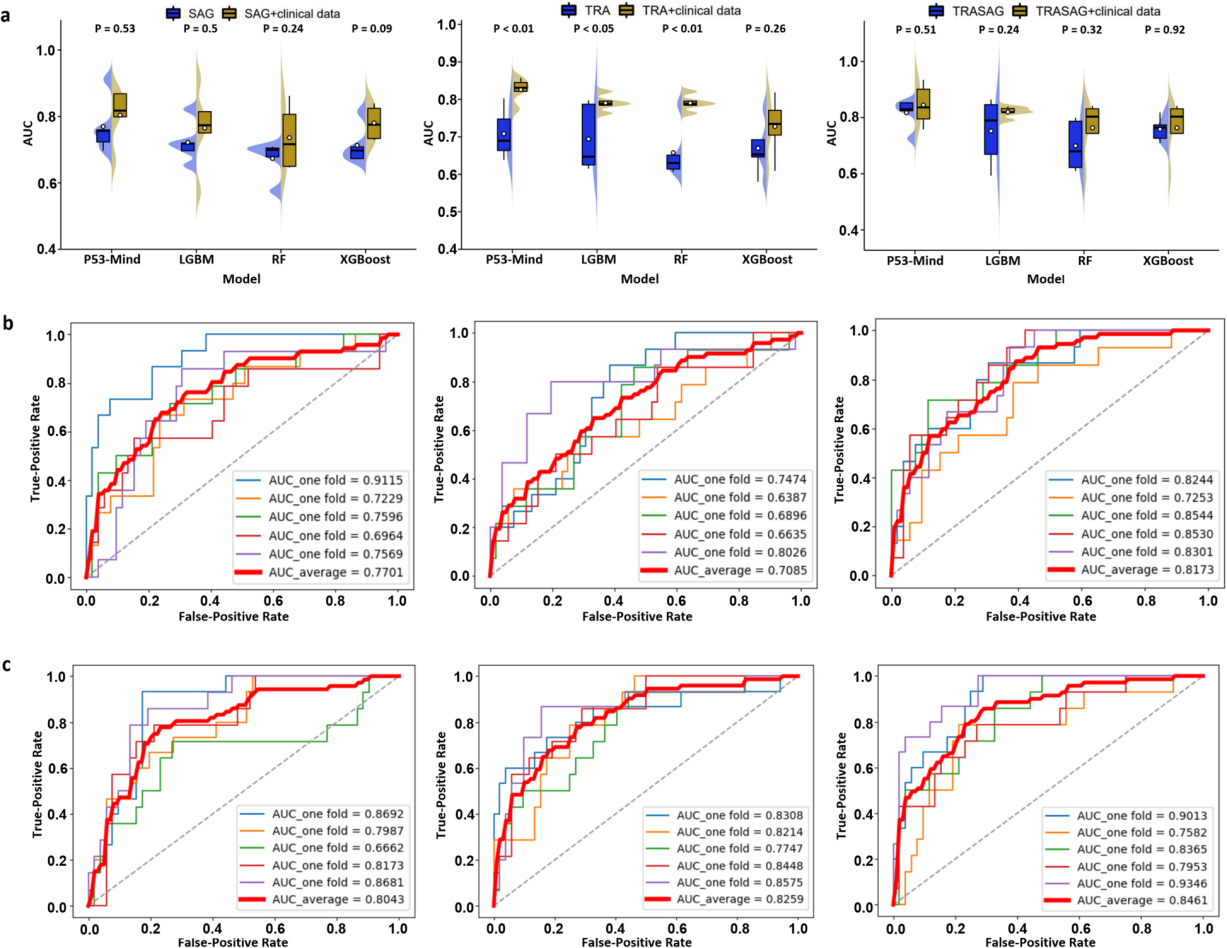
The fivefold cross-validation results in the *P53* genotype prediction task. **a** The comparison results of the proposed P53-Mind with the mainstream machine learning models. **b** The ROC curves of P53-Mind when inputting features of the SAG phase (left), TRA phase (middle), and SAG combined with TRA phase (right). **c** The ROC curves of P53-Mind when the features of the SAG phase-clinical baseline (left), TRA phase-clinical baseline (middle), and SAG phase-TRA phase-clinical baseline (right) are inputted. AUC, area under the curve; P53, tumor protein p53; ROC, receiver operating characteristic; SAG, sagittal; TRA, transverse

Four machine learning models were trained for each prediction task, and each model was fed into six feature representations. All models were parameter optimized in the experiments, which facilitated the performance comparison. The results of the WHO tumor grade prediction and ROC curves of the proposed models are shown in Fig. 3. Among the four models, WHO-Mind obtained the best average area under the ROC curve (AUC; 0.9263), with the AUC higher than that of XGBoost (0.8802), LightGBM (0.9079), and RF (0.8618), indicating the superiority of the neural network architecture in this prediction task. However, inputting radiomics features combined with clinical baseline features was superior to only inputting radiomics features, and the best-performing combination was SAG + TRA + clinical indicators. Notably, we found similar findings in performing the prediction task of *ATRX* and *P53* mutation status, which may be due to the large variety of features covered by multimodal fusion. The results of the *ATRX* and *P53* mutation predictions for each model are shown in Figs. 4 and 5, respectively. The proposed ATRX-Mind and P53-Mind models performed better than the compared models, where the highest AUCs were 0.9281 and 0.9173, respectively. To reflect the comprehensiveness of the evaluation of the proposed models, their Acc, Sens, Spec, and *F*_1_ scores were also calculated and summarized in Additional file 9.

### Results of prediction models in the external test cohort

**Fig. 6:**
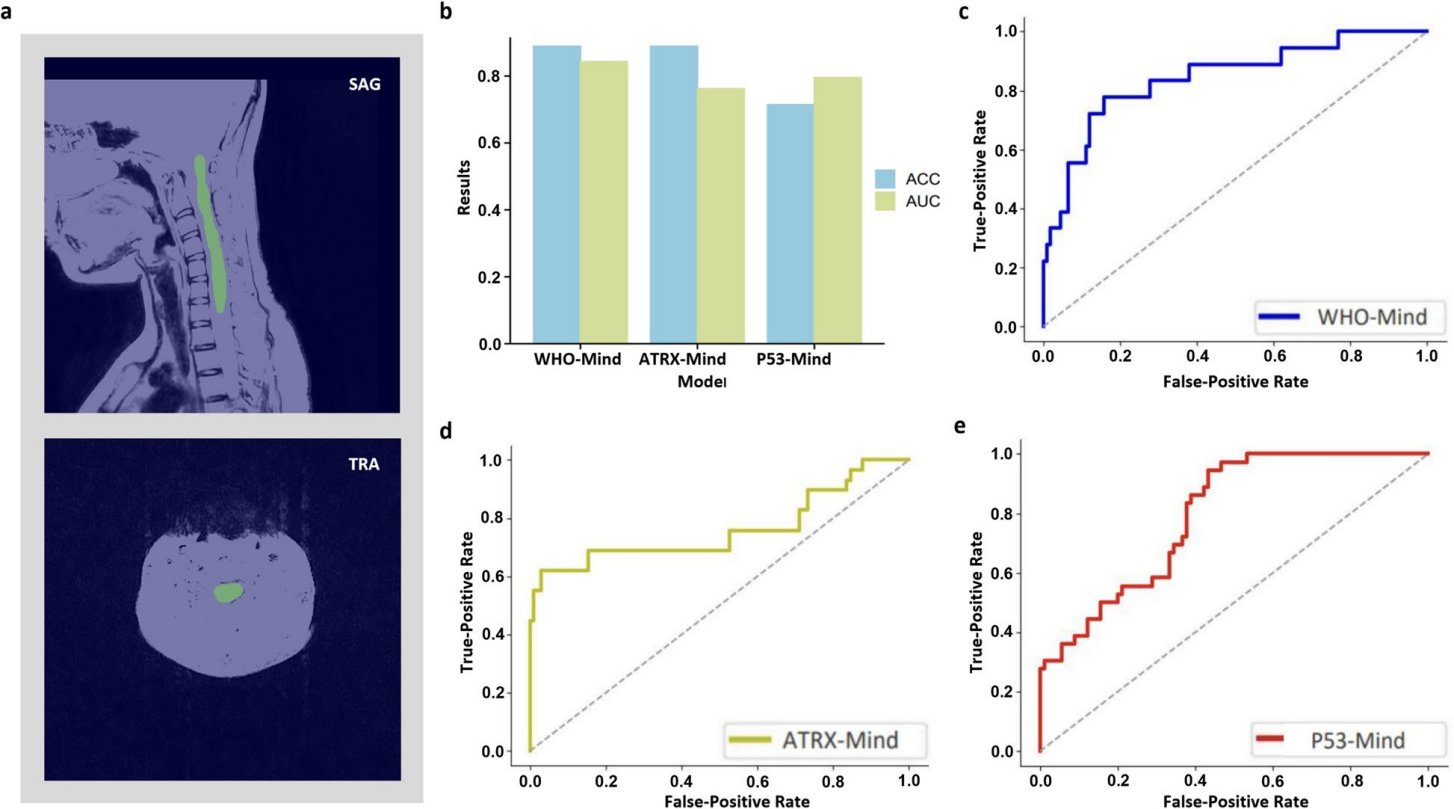
Results of external validation using the proposed models. **a** The visualization of lesion segmentation using the automatic segmentation model in SAG and TRA phases, respectively. **b** The external validation results adopting the proposed models in different tasks. **c**–**e** The ROC curves for each model in the prediction tasks. ATRX, alpha thalassemia/mental retardation syndrome X-linked; ROC, receiver operating characteristic; P53, tumor protein p53; SAG, sagittal; TRA, transverse; WHO, World Health Organization

The best-performing models (i.e., WHO-Mind, ATRX-Mind, and P53-Mind) were selected for external testing. Multimodal fused feature representations were fed into each model, namely, the SAG + TRA + clinical indicators. The automatic segmentation effect of the Swin transformer is shown in Fig. 6a. This was found to segment the lesion area with relative accuracy. The performance of the models in each test task is shown in Fig. 6b. WHO-Mind reached the highest AUC (0.8431), and both WHO-Mind and ATRX-Mind obtained the highest Acc (0.8889). In addition, the ROCs of the three models were plotted to visualize their generalization performance in more detail (Fig. 6c–e). More detailed test results (e.g., Acc, Sens, Spec, and *F*_1_ scores) are displayed in Additional file 10.

## Discussion

Biopsy to obtain tumor tissue for histological examination in spinal cord tumors is dangerous and not worthwhile because of the high risk. In this study, we developed three models that can segment the IMG region automatically and predict the *ATRX*/*P53* mutation status and the grade of IMGs based on preoperative sagittal and transverse MRI scans. The prediction models offer a non-invasive alternative for identifying the tumor grade and histopathological characteristics in patients with IMGs.

Many studies have used machine learning methods to identify the genetic and histological features of brain gliomas [6, 7]. Li et al. [25] adopted a support vector machine model to predict the genetic characteristics of *ATRX*. Given that their method is only effective for low-grade gliomas, the tumor grade should be known in advance, complicating the use of their method in the preoperative period. Gao et al. [7] used multiple machine learning-based radiomic approaches to predict the brain glioma grade and status of pathologic biomarkers, including Ki-67, GFAP, and S100. However, these biomarkers are interrelated and are not highly specific to gliomas, according to the WHO CNS5. In real-world clinical settings, it is not possible to accurately determine the type of glioma preoperatively. Therefore, models must address all types of gliomas, including WHO grades I–IV. As such, this study consecutively enrolled all types of spinal cord gliomas from two centers.

Many studies have focused on predicting *IDH* mutation and 1p/19q co-deletion status [6]. Although *IDH* mutation and 1p/19q co-deletion status are important biomarkers for the prognosis and survival of gliomas, we did not predict this in our study. On the one hand, *IDH* mutation in IMGs is not as common as in brain gliomas and cannot be used to distinguish between grade I pilocytic astrocytoma and grade II diffuse astrocytoma. On the other hand, *IDH* IHC would be ineffective because most IMGs lack the conventional *IDH1* p.R132H mutation [12]. Furthermore, diffuse astrocytomas with *IDH* mutations are most likely to harbor mutations in *ATRX* and *P53* [26], and cIMPACT-NOW Update 2 states that a definite loss of ATRX nuclear expression and/or p53 immunopositivity is sufficient for the diagnosis of astrocytoma without the need for a 1p/19q test [27]. Therefore, *ATRX* and *P53* mutations are more useful for identifying IMGs and are also important clinical behavior indicators. Our models, focusing on *ATRX* and *P53* mutation status, will bring significant convenience in diagnosing IMGs.

Traditional lesion segmentation is performed manually, which is time-consuming and labor-intensive. Progress in the automatic segmentation of gliomas has been reported more frequently [28–31], but only few studies have attempted to segment spinal cord tumor automatically due to the lack of sufficient training in such rare tumors [8, 10]. This study employed a deep learning strategy based on transformer architecture to segment IMGs, and relatively satisfactory segmentation results were obtained (the DSC was 0.8697 for SAG and 0.8738 for TRA). To our best knowledge, this study is the first to employ such a strategy. Although the segmentation results temporarily have a certain gap compared with manual annotation, the method significantly improves efficiency and facilitates end-to-end prediction. We will further improve this algorithm to achieve more accurate automatic segmentation.

Additionally, model performance was compared using different combinations of SAG images, TRA images, and clinical features. The combination of SAG and TRA features exhibited a better discriminatory ability than either feature alone, probably because SAG and TRA features can form some complementary properties. Most IMGs are confined to the spinal cord and grow along the long axis of the spinal cord. SAG images can show the greatest anatomical detail and other tumor-related lesions, such as the spinal cord cavity and edema. However, only a few scanning slices are obtained from SAG T2WI, typically 11 slices, which is insufficient for constructing 3D tumor models. TRA T2WI scans usually have smaller slice spacing, have the advantage of accurately constructing a 3D tumor ROI, and may allow for more accurate delineation of the tumor contour during segmentation. Additionally, previous research has confirmed that clinical features such as age and sex are related to glioma type [32, 33]. Wu et al. [34] developed a nomogram strategy to adopt clinical features in radiomics-based prediction models. Fifteen clinical baseline features, including seven baseline features and eight radiological signs that are difficult to represent with the radiomic approach, selected by univariate regression and prior clinical experience were adopted in our models. Although the SAG + TRA radiomics input had already achieved satisfactory performance, the model showed better differentiation ability when we added clinical features.

It is important to emphasize that the three proposed neural networks showed superior performance to the mainstream ensemble learning models in all prediction tasks, which was encouraging. This may be related to the models’ architecture. Specifically, the neural networks proposed in this study underwent extensive pre-experimentation, including setting the number of layers and the number of neurons. External tests were also performed to verify their generalizability, and the results were satisfactory. For the WHO tumor grade, *ATRX* mutation status, and *P53* mutation status prediction tasks, the proposed model obtained AUCs of 0.8431, 0.7622, and 0.7954, respectively, in the external validation set.

Our study has some limitations. First, we selected only the commonly used ATRX and P53 biomarkers for IMGs from a broad range of biomarkers. The detection of other important biomarkers in IMGs, such as Ki-67 and H3 K27M, may also benefit from an automatic machine learning approach. Second, our models should input more patient characteristics in the future to allow for a more comprehensive assessment of patient conditions. For example, our models still need input with respect to some radiological characteristics, such as the spinal cord cavity, edema, and atrophy, which require a priori knowledge assessment by radiologists. Multiple imaging inputs, including normal spinal cord and tumor-related lesions, may help to extract all radiological characteristics automatically. Third, although our data originated from two institutes with one for external validation, a larger dataset from multiple institutes is needed to balance the heterogeneity in different MR-acquiring machines. Finally, there is a degree of imbalance in the proportion of data of different types (e.g., different grades or mutation states) in this study. The dataset should be balanced in the future.

## Conclusions

We developed the first machine learning strategy to predict IMG grade and *ATRX* and *P53* mutation status while simultaneously automatically segmenting the tumor. Neural networks based on multimodal fusion (i.e., the SAG phase-TRA phase-clinical baseline) were shown to have the best prediction performance. Although further validation must be performed in more institutes before widespread clinical application, we believe this research is a significant attempt at the automatic diagnosis of IMGs.

## Supplementary Information

**Additional file 1.** MRI parameters per manufacturer. Three MRI scanners, namely, GE, Siemens and Philips, were used in this study. Each machine has different scanning parameters for sagittal and transverse T2-weighted images of the cervical, thoracic, and lumbar vertebrae. This table lists the important MRI scanning parameters before image preprocessing.

**Additional file 2.** Architecture of the proposed models and optimized key hyperparameter settings.

**Additional file 3.** Clinical characteristics of the patients and distribution of the ATRX and P53 mutation status across the primary and external validation cohorts. ATRX, alpha thalassemia/mental retardation syndrome X-linked; IQR, interquartile range; P53, tumor protein p53; WHO, World Health Organization.

**Additional file 4.** Selected stable features in TRA and SAG images. To avoid interobserver variations during manual segmentation, we calculated the intraclass correlation coefficientfor each feature, and only those with high stabilitywere included in the analysis. The above table shows the proportion of stable features in each feature class extracted from TRA and SAG images. GLDM, gray-level dependence matrix; GLRLM, gray-level run-length matrix; GLSZM, gray-level size zone matrix; NGTDM, neighboring gray-tone difference matrix; SAG, sagittal; TRA, transverse.

**Additional file 5.** Feature selection with LASSO in the WHO grading task.Feature selection in the SAG group. Twenty-four features with non-zero coefficients were selected using the minimum criteria.LASSO coefficient profiles of the features in the SAG group. Each colored line represents the coefficient of each feature.Feature selection in the TRA group. Six features with non-zero coefficients were selected using the minimum criteria.LASSO coefficient profiles of the features in the TRA group.Feature selection in the SAG+TRA group. Twenty features with non-zero coefficients were selected using the minimum criteria.LASSO coefficient profiles of the features in the SAG+TRA group. LASSO, least absolute shrinkage and selection operator; SAG, sagittal; TRA, transverse; WHO, World Health Organization.

**Additional file 6.** Feature selection with LASSO in the ATRX task.Feature selection in the SAG group. Three features with non-zero coefficients were selected using the minimum criteria.LASSO coefficient profiles of the features in the SAG group. Each colored line represents the coefficient of each feature.Feature selection in the TRA group. Thirty-nine features with non-zero coefficients were selected using the minimum criteria.LASSO coefficient profiles of the features in the TRA group.Feature selection in the SAG+TRA group. Five features with non-zero coefficients were selected using the minimum criteria.LASSO coefficient profiles of the features in the SAG+TRA group. ATRX, alpha thalassemia/mental retardation syndrome X-linked, LASSO, least absolute shrinkage and selection operator; SAG, sagittal; TRA, transverse.

**Additional file 7.** Feature selection with LASSO in the P53 task.Feature selection in the SAG group. Twenty-one features with non-zero coefficients were selected using the minimum criteria.LASSO coefficient profiles of the features in the SAG group. Each colored line represents the coefficient of each feature.Feature selection in the TRA group. Twenty-four features with non-zero coefficients were selected using the minimum criteria.LASSO coefficient profiles of the features in the TRA group.Feature selection in the SAG+TRA group. Fifty-seven features with non-zero coefficients were selected using the minimum criteria.LASSO coefficient profiles of the features in the SAG+TRA group. LASSO, least absolute shrinkage and selection operator; P53, tumor protein p53; SAG, sagittal; TRA, transverse.

**Additional file 8.** Selected radiomics features in each prediction task. In the WHO tumor grade prediction task, 24 features in SAG, 6 features in TRA, and 20 features in SAG+TRA were selected. In the ATRX prediction task, 3 features in SAG, 39 features in TRA, and 5 features in SAG+TRA were selected. In the P53 prediction task, 21 features in SAG, 24 features in TRA, and 57 features in SAG+TRA were selected. SAG, sagittal; TRA, transverse.

**Additional file 9.** Detailed results of the 5-fold cross-validation for the proposed models using multimodal fusion features in the primary cohort. Acc: accuracy, Sens: sensitivity, Spec: specificity. The above results are expressed using the mean of 5 experiments and the corresponding 95% confidence interval.

**Additional file 10.** Detailed test results in the external test cohort. Acc: accuracy, Sens: sensitivity, Spec: specificity.

## Data Availability

The datasets used and/or analyzed during the current study are available from the corresponding author upon reasonable request.

## Abbreviations

Acc: Accuracy
ATRX: Alpha thalassemia/mental retardation syndrome X-linked
DNN: Deep neural network
DSC: Dice similarity coefficient
ICC: Intraclass correlation coefficient
IDH: Isocitrate dehydrogenase
IHC: Immunohistochemistry
IMG: Intramedullary glioma
LASSO: Least absolute shrinkage and selection operator
LightGBM: Gradient Boosting Decision Tree
MRI: Magnetic resonance imaging
P53: Tumor protein p53
RF: Random forest
ROC: Receiver operating characteristic
ROI: Region of interest
SAG: Sagittal
T2WI: T2-weighted imaging
TRA: Transverse
XGBoost: Extreme Gradient Boosting
