# Supplementary material for: Multimodal-based machine learning strategy for accurate and non-invasive prediction of intramedullary glioma grade and mutation status of molecular markers: a retrospective study
Source: BMC Med. 2023 May 29;21:198. doi: 10.1186/s12916-023-02898-4 (PMC10228074; doi:10.1186/s12916-023-02898-4)
Supplement: Supplementary file 1 — Additional file 1. MRI parameters per manufacturer. Three MRI scanners, namely, GE, Siemens and Philips, were used in this study. Each machine has different scanning parameters for sagittal and transverse T2-weighted images of the cervical, thoracic, and lumbar vertebrae. This table lists the important MRI scanning parameters before image preprocessing. [file 12916_2023_2898_MOESM1_ESM.docx]

**Additional file 1. MRI parameters per manufacturer**

| Manufacturer | GE | | Siemens | | Philips | |
| --- | --- | --- | --- | --- | --- | --- |
| Sequence | T2TRA | T2SAG | T2TRA | T2SAG | T2TRA | T2SAG |
| Repetition time (ms) | 3000–9400 | 2500–4600 | 3000–6740 | 2200–4300 | 2100–5000 | 1800–3000 |
| Echo time (ms) | 65–128 | 97–129 | 70–143 | 78–117 | 90, 120 | 80, 90, 95, 100 |
| Slice thickness (mm) | 2–7 | 3, 3.5, or 4 | 4, 5, 5.5, 6 | 3–4.5 | 4, 5, 5.5, 6 | 3–5.5 |
| Flip angle (°) | 142 | 142 | 90–180 | 120, 150, or 160 | 90 | 90 |
| Matrix size | 512×512 | 512×512 | 320×320 or  384×384 | 256×256, 320×320, or  384×384 | 400×400, 432×432, or  448×448 | 384×384–800×800 |
| Slices | 15–34 | 11–13 | 10–34 | 11–13 | 10–36 | 11–13 |
| Spacing between slices (mm) | 2.5–9 | 3.8 or 5 | 3.2–9.1 | 3.3–5.75 | 4.4–7 | 3.3–6 |
| Pixel spacing (mm) | 0.355–0.560 | 0.468–0.664 | 0.563–0.875 | 0.729–1.328 | 0.335–0.5 | 0.411–0.661 |

Three MRI scanners, GE, Siemens, and Philips, were used in this study. Each machine has different scanning parameters for sagittal and transverse T2-weighted images of the cervical, thoracic, and lumbar vertebrae; this table lists the important MRI scanning parameters before image preprocessing.

SAG, sagittal; TRA, transverse; MRI, magnetic resonance imaging
