# Supplementary material for: Multimodal-based machine learning strategy for accurate and non-invasive prediction of intramedullary glioma grade and mutation status of molecular markers: a retrospective study
Source: BMC Med. 2023 May 29;21:198. doi: 10.1186/s12916-023-02898-4 (PMC10228074; doi:10.1186/s12916-023-02898-4)
Supplement: Supplementary file 2 — Additional file 2. Architecture of the proposed models and optimized key hyperparameter settings. [file 12916_2023_2898_MOESM2_ESM.docx]

**Additional file 2. Architecture of the proposed models and optimized key hyperparameter settings**

| Deep Neural Network | Input Layer | Hidden Layers | Output Layer | Activation Function | Optimizer | Initial Learning Rate | Batch Size |
| --- | --- | --- | --- | --- | --- | --- | --- |
| WHO-Mind | 1 | 4 | 1 | ReLU | Adam | 0.01 | 64 |
| ATRX-Mind | 1 | 3 | 1 | ReLU | Adam | 0.01 | 64 |
| P53-Mind | 1 | 3 | 1 | ReLU | Adam | 0.01 | 64 |
