# Supplementary material for: Multimodal-based machine learning strategy for accurate and non-invasive prediction of intramedullary glioma grade and mutation status of molecular markers: a retrospective study
Source: BMC Med. 2023 May 29;21:198. doi: 10.1186/s12916-023-02898-4 (PMC10228074; doi:10.1186/s12916-023-02898-4)
Supplement: Supplementary file 3 — Additional file 3. Clinical characteristics of the patients and distribution of the ATRX and P53 mutation status across the primary and external validation cohorts. ATRX, alpha thalassemia/mental retardation syndrome X-linked; IQR, interquartile range; P53, tumor protein p53; WHO, World Health Organization. [file 12916_2023_2898_MOESM3_ESM.docx]

**Additional file 3. Clinical characteristics of patients and the distribution of the *ATRX* and *P53* mutation status across the primary and external validation cohorts**

| Characteristic | Primary cohort (n=332) | External validation cohort (n=127) | p |
| --- | --- | --- | --- |
| Age (years), median (IQR) | 37 (29, 51) | 41 (31.5, 52.5) | 0.274 |
| Sex, n (%) |  |  | 0.265 |
| Female | 113 (34%) | 51 (40.2%) |  |
| Male | 219 (66%) | 76 (59.8%) |  |
| WHO, n (%) |  |  | 0.001 |
| I | 70 (21.1%) | 8 (6.3%) |  |
| II | 210 (63.3%) | 98 (77.2%) |  |
| III | 35 (10.5%) | 11 (8.7%) |  |
| IV | 17 (5.1%) | 10 (7.9%) |  |
| Glioma grade, n (%) |  |  | 0.931 |
| High grade (WHO III-IV) | 52 (15.7%) | 21 (16.5%) |  |
| Low grade (WHO I-II) | 280 (84.3%) | 106 (83.5%) |  |
| P53, n (%) |  |  | 0.006 |
| 0 | 260 (78.3%) | 83 (65.4%) |  |
| 1 | 72 (21.7%) | 44 (34.6%) |  |
| ATRX, n (%) |  |  | 0.039 |
| 0 | 253 (76.2%) | 84 (66.1%) |  |
| 1 | 79 (23.8%) | 43 (33.9%) |  |
| Time of onset (months), median (IQR) | 24 (5, 48) | 24 (3, 48) | 1.000 |
| Accompanying diseases, n (%) |  |  | 0.890 |
| 0 | 205 (61.7%) | 80 (63%) |  |
| 1 | 127 (38.3%) | 47 (37%) |  |
| Smoking, n (%) |  |  |  |
| 0 | 303 (91.3%) | 113 (89%) | 0.566 |
| 1 | 29 (8.7%) | 14 (11%) |  |
| Alcohol, n (%) |  |  | 0.490 |
| 0 | 318 (95.8%) | 119 (93.7%) |  |
| 1 | 14 (4.2%) | 8 (6.3%) |  |
| McCormick, n (%) |  |  | 0.848 |
| 1 | 157 (47.3%) | 59 (46.5%) |  |
| 2 | 103 (31%) | 44 (34.6%) |  |
| 3 | 30 (9%) | 10 (7.9%) |  |
| 4 | 42 (12.7%) | 14 (11.0%) |  |
| Single_tumor, n (%) |  |  | 0.268 |
| 0 | 22 (6.6%) | 13 (10.2%) |  |
| 1 | 310 (93.4%) | 114 (89.8%) |  |
| Axis ratio (tumor/spinal cord), n (%) |  |  | <0.001 |
| <25 | 5 (1.5%) | 0 (0%) |  |
| 25–50 | 5 (1.5%) | 0 (0%) |  |
| 50–75 | 59 (17.8%) | 3 (2.4%) |  |
| 75–100 | 263 (79.2%) | 124 (97.6%) |  |
| Tumor bleeding, n (%) |  |  | <0.001 |
| 0 | 243 (73.2%) | 118 (92.9%) |  |
| 1 | 89 (26.8%) | 9 (7.1%) |  |
| Tumor cysts, n (%) |  |  | 0.641 |
| 0 | 185 (55.7%) | 67 (52.8%) |  |
| 1 | 147 (44.3%) | 60 (47.2%) |  |
| Spinal cord cavity, n (%) |  |  | 0.051 |
| 0 | 215 (64.8%) | 69 (54.3%) |  |
| 1 | 117 (35.2%) | 58 (45.7%) |  |
| Spinal cord edema, n (%) |  |  | 0.771 |
| 0 | 188 (56.6%) | 70 (55.1%) |  |
| 1 | 144 (43.4%) | 57 (44.9%) |  |
| Spinal cord atrophy, n (%) |  |  | 0.320 |
| 0 | 228 (68.7%) | 99 (78.0%) |  |
| 1 | 104 (31.3%) | 28 (22.0%) |  |
| Spine malformation, n (%) |  |  | 1.000 |
| 0 | 289 (87%) | 111 (87.4%) |  |
| 1 | 43 (13%) | 16 (12.6%) |  |

ATRX, alpha thalassemia/mental retardation syndrome X-linked; IQR, interquartile range; P53, tumor protein p53; WHO, World Health Organization
