# Supplementary material for: Multimodal-based machine learning strategy for accurate and non-invasive prediction of intramedullary glioma grade and mutation status of molecular markers: a retrospective study
Source: BMC Med. 2023 May 29;21:198. doi: 10.1186/s12916-023-02898-4 (PMC10228074; doi:10.1186/s12916-023-02898-4)
Supplement: Supplementary file 4 — Additional file 4. Selected stable features in TRA and SAG images. To avoid interobserver variations during manual segmentation, we calculated the intraclass correlation coefficientfor each feature, and only those with high stabilitywere included in the analysis. The above table shows the proportion of stable features in each feature class extracted from TRA and SAG images. GLDM, gray-level dependence matrix; GLRLM, gray-level run-length matrix; GLSZM, gray-level size zone matrix; NGTDM, neighboring gray-tone difference matrix; SAG, sagittal; TRA, transverse. [file 12916_2023_2898_MOESM4_ESM.docx]

**Additional file 4. Selected stable features in TRA and SAG images**

|  | First order | Shape | GLRLM | GLSZM | NGTDM | GLDM | Sum |
| --- | --- | --- | --- | --- | --- | --- | --- |
| TRA | 206/252 | 14/14 | 196/224 | 202/224 | 55/70 | 177/196 | 850/980 (86.7%) |
| SAG | 131/252 | 14/14 | 181/224 | 180/224 | 47/70 | 169/196 | 722/980 (73.7%) |
| Sum | 338/504 (67.1%) | 28/28 (100.0%) | 337/448 (84.2%) | 382/448 (85.3%) | 102/140 (85.9%) | 346/392 (72.9%) | 1572/1960 (80.1%) |

To avoid interobserver variations during manual segmentation, we calculated the intraclass correlation coefficient (ICC) for each feature, and only those with high stability (ICC > 0.8) were included in the analysis. The above table shows the proportion of stable features in each feature class extracted from TRA and SAG images.

GLDM, gray-level dependence matrix; GLRLM, gray-level run-length matrix; GLSZM, gray-level size zone matrix; NGTDM, neighboring gray-tone difference matrix; SAG, sagittal; TRA, transverse
