# Supplementary material for: Multimodal-based machine learning strategy for accurate and non-invasive prediction of intramedullary glioma grade and mutation status of molecular markers: a retrospective study
Source: BMC Med. 2023 May 29;21:198. doi: 10.1186/s12916-023-02898-4 (PMC10228074; doi:10.1186/s12916-023-02898-4)
Supplement: Supplementary file 6 — Additional file 6. Feature selection with LASSO in the ATRX task.Feature selection in the SAG group. Three features with non-zero coefficients were selected using the minimum criteria.LASSO coefficient profiles of the features in the SAG group. Each colored line represents the coefficient of each feature.Feature selection in the TRA group. Thirty-nine features with non-zero coefficients were selected using the minimum criteria.LASSO coefficient profiles of the features in the TRA group.Feature selection in the SAG+TRA group. Five features with non-zero coefficients were selected using the minimum criteria.LASSO coefficient profiles of the features in the SAG+TRA group. ATRX, alpha thalassemia/mental retardation syndrome X-linked, LASSO, least absolute shrinkage and selection operator; SAG, sagittal; TRA, transverse. [file 12916_2023_2898_MOESM6_ESM.docx]

**Additional file 6. Feature selection with LASSO in the ATRX task**


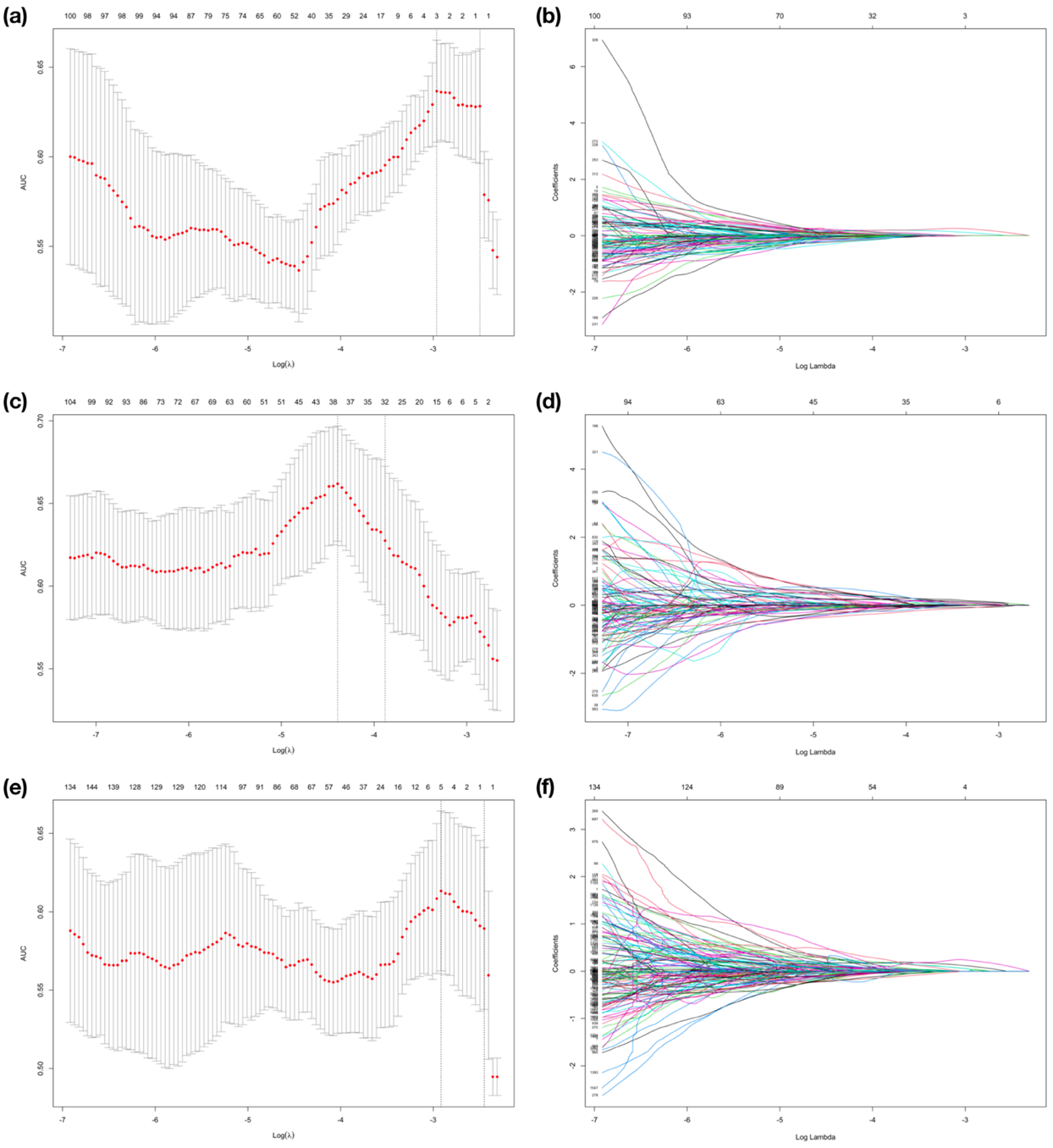


(**a**) Feature selection in the SAG group. Three features with non-zero coeﬃcients were selected using the minimum criteria. (**b**) LASSO coeﬃcient proﬁles of the features in the SAG group. Each colored line represents the coeﬃcient of each feature. (**c**) Feature selection in the TRA group. Thirty-nine features with non-zero coeﬃcients were selected using the minimum criteria. (**d**) LASSO coeﬃcient proﬁles of the features in the TRA group. (**e**) Feature selection in the SAG+TRA group. Five features with non-zero coeﬃcients were selected using the minimum criteria. (**f**) LASSO coeﬃcient proﬁles of the features in the SAG+TRA group. ATRX, alpha thalassemia/mental retardation syndrome X-linked, LASSO, least absolute shrinkage and selection operator; SAG, sagittal; TRA, transverse
