# Supplementary material for: Multimodal-based machine learning strategy for accurate and non-invasive prediction of intramedullary glioma grade and mutation status of molecular markers: a retrospective study
Source: BMC Med. 2023 May 29;21:198. doi: 10.1186/s12916-023-02898-4 (PMC10228074; doi:10.1186/s12916-023-02898-4)
Supplement: Supplementary file 8 — Additional file 8. Selected radiomics features in each prediction task. In the WHO tumor grade prediction task, 24 features in SAG, 6 features in TRA, and 20 features in SAG+TRA were selected. In the ATRX prediction task, 3 features in SAG, 39 features in TRA, and 5 features in SAG+TRA were selected. In the P53 prediction task, 21 features in SAG, 24 features in TRA, and 57 features in SAG+TRA were selected. SAG, sagittal; TRA, transverse. [file 12916_2023_2898_MOESM8_ESM.docx]

**Additional file 8. Selected radiomics features in each prediction task**

| Selected features in the WHO tumor grade task | |
| --- | --- |
| SAG feature group  (n=24) | \| SAG_original_shape_Flatness \| \| --- \| \| SAG_original_shape_MajorAxisLength \| \| SAG_original_shape_Maximum2DDiameterRow \| \| SAG_wavelet.LHL_firstorder_90Percentile \| \| SAG_wavelet.LHL_firstorder_Skewness \| \| SAG_wavelet.LHH_firstorder_Kurtosis \| \| SAG_wavelet.HLL_firstorder_InterquartileRange \| \| SAG_wavelet.HLL_glszm_ZonePercentage \| \| SAG_wavelet.HLL_gldm_SmallDependenceEmphasis \| \| SAG_wavelet.HLL_ngtdm_Contrast \| \| SAG_wavelet.HLH_firstorder_Skewness \| \| SAG_wavelet.HLH_glszm_LargeAreaHighGrayLevelEmphasis \| \| SAG_wavelet.HLH_glszm_SmallAreaLowGrayLevelEmphasis \| \| SAG_wavelet.HHL_ngtdm_Contrast \| \| SAG_wavelet.LLL_firstorder_RobustMeanAbsoluteDeviation \| \| SAG_logarithm_firstorder_InterquartileRange \| \| SAG_logarithm_firstorder_RootMeanSquared \| \| SAG_logarithm_glrlm_ShortRunHighGrayLevelEmphasis \| \| SAG_logarithm_glrlm_ShortRunLowGrayLevelEmphasis \| \| SAG_logarithm_ngtdm_Busyness \| \| SAG_logarithm_ngtdm_Contrast \| \| SAG_exponential_firstorder_Skewness \| \| SAG_gradient_firstorder_Median \| \| SAG_gradient_firstorder_Skewness \| |
| TRA feature group  (n=6) | \| TRA_wavelet.LLH_glszm_SizeZoneNonUniformityNormalized \| \| --- \| \| TRA_wavelet.LLH_glszm_SmallAreaEmphasis \| \| TRA_wavelet.LHL_firstorder_Skewness \| \| TRA_wavelet.LHH_ngtdm_Contrast \| \| TRA_wavelet.HLL_glszm_ZoneEntropy \| \| TRA_logarithm_firstorder_Maximum \| |
| SAG+TRA feature group  (n=20) | \| TRA_wavelet.LLH_glszm_SmallAreaEmphasis \| \| --- \| \| TRA_wavelet.LHL_firstorder_Skewness \| \| TRA_wavelet.LHH_ngtdm_Contrast \| \| TRA_wavelet.HLL_glszm_ZoneEntropy \| \| TRA_logarithm_firstorder_Maximum \| \| SAG_original_shape_Flatness \| \| SAG_original_shape_MajorAxisLength \| \| SAG_original_shape_Maximum2DDiameterRow \| \| SAG_wavelet.LHL_firstorder_Skewness \| \| SAG_wavelet.LHH_firstorder_Kurtosis \| \| SAG_wavelet.HLL_firstorder_InterquartileRange \| \| SAG_wavelet.HLL_gldm_SmallDependenceEmphasis \| \| SAG_wavelet.HLL_ngtdm_Contrast \| \| SAG_wavelet.HLH_firstorder_Skewness \| \| SAG_wavelet.HLH_glszm_SmallAreaLowGrayLevelEmphasis \| \| SAG_wavelet.LLL_firstorder_InterquartileRange \| \| SAG_logarithm_firstorder_RootMeanSquared \| \| SAG_logarithm_ngtdm_Contrast \| \| SAG_gradient_firstorder_Median \| \| SAG_gradient_firstorder_Skewness \| |
|  | Selected features in the ATRX task |
| SAG feature group  (n=3) | \| SAG_wavelet.HLL_firstorder_InterquartileRange \| \| --- \| \| SAG_wavelet.HLH_glszm_SizeZoneNonUniformityNormalized \| \| SAG_wavelet.HLH_gldm_DependenceNonUniformityNormalized \| |
| TRA feature group  (n=39) | \| TRA_original_shape_MajorAxisLength \| \| --- \| \| TRA_original_glszm_LargeAreaEmphasis \| \| TRA_original_glszm_LargeAreaHighGrayLevelEmphasis \| \| TRA_original_ngtdm_Complexity \| \| TRA_wavelet.LLH_glszm_ZoneEntropy \| \| TRA_wavelet.LLH_gldm_DependenceNonUniformityNormalized \| \| TRA_wavelet.LHL_firstorder_Minimum \| \| TRA_wavelet.LHL_glszm_LargeAreaEmphasis \| \| TRA_wavelet.LHL_glszm_SmallAreaLowGrayLevelEmphasis \| \| TRA_wavelet.LHL_glszm_ZoneVariance \| \| TRA_wavelet.LHL_gldm_DependenceNonUniformityNormalized \| \| TRA_wavelet.LHL_gldm_DependenceVariance \| \| TRA_wavelet.LHL_gldm_LargeDependenceHighGrayLevelEmphasis \| \| TRA_wavelet.LHH_firstorder_Maximum \| \| TRA_wavelet.LHH_glrlm_LongRunLowGrayLevelEmphasis \| \| TRA_wavelet.LHH_glszm_SmallAreaEmphasis \| \| TRA_wavelet.LHH_ngtdm_Contrast \| \| TRA_wavelet.HLL_firstorder_Skewness \| \| TRA_wavelet.HLL_glrlm_RunVariance \| \| TRA_wavelet.HLL_glszm_GrayLevelNonUniformityNormalized \| \| TRA_wavelet.HLL_glszm_ZoneEntropy \| \| TRA_wavelet.HLH_glszm_SizeZoneNonUniformity \| \| TRA_wavelet.HHL_gldm_SmallDependenceHighGrayLevelEmphasis \| \| TRA_wavelet.HHH_glszm_SmallAreaLowGrayLevelEmphasis \| \| TRA_wavelet.LLL_glszm_LargeAreaEmphasis \| \| TRA_wavelet.LLL_glszm_LargeAreaLowGrayLevelEmphasis \| \| TRA_wavelet.LLL_ngtdm_Busyness \| \| TRA_square_glszm_LargeAreaEmphasis \| \| TRA_square_glszm_SmallAreaLowGrayLevelEmphasis \| \| TRA_square_gldm_DependenceVariance \| \| TRA_squareroot_glszm_LargeAreaEmphasis \| \| TRA_squareroot_ngtdm_Busyness \| \| TRA_logarithm_firstorder_RootMeanSquared \| \| TRA_logarithm_glszm_LargeAreaEmphasis \| \| TRA_logarithm_ngtdm_Busyness \| \| TRA_exponential_glszm_LargeAreaEmphasis \| \| TRA_exponential_glszm_LargeAreaHighGrayLevelEmphasis \| \| TRA_exponential_glszm_LargeAreaLowGrayLevelEmphasis \| |
| SAG+TRA feature group  (n=5) | \| TRA_wavelet.LHH_glrlm_LongRunLowGrayLevelEmphasis \| \| --- \| \| TRA_wavelet.HLL_glrlm_LongRunEmphasis \| \| TRA_wavelet.HLL_glrlm_RunVariance \| \| SAG_wavelet.HLL_firstorder_InterquartileRange \| \| SAG_wavelet.HLH_glszm_SizeZoneNonUniformityNormalized \| |
| Selected features in the P53 task | |
| SAG feature group  (n=21) | \| SAG_original_shape_Flatness \| \| --- \| \| SAG_original_shape_MinorAxisLength \| \| SAG_wavelet.LLH_firstorder_Skewness \| \| SAG_wavelet.LLH_glrlm_ShortRunLowGrayLevelEmphasis \| \| SAG_wavelet.LLH_glszm_GrayLevelNonUniformityNormalized \| \| SAG_wavelet.HLL_firstorder_InterquartileRange \| \| SAG_wavelet.HLL_glrlm_GrayLevelNonUniformityNormalized \| \| SAG_wavelet.HLL_glrlm_LongRunLowGrayLevelEmphasis \| \| SAG_wavelet.HLL_glszm_SizeZoneNonUniformityNormalized \| \| SAG_wavelet.HLL_gldm_LargeDependenceHighGrayLevelEmphasis \| \| SAG_wavelet.HLH_firstorder_Skewness \| \| SAG_wavelet.HLH_glszm_ZoneEntropy \| \| SAG_wavelet.HHL_firstorder_Kurtosis \| \| SAG_wavelet.HHL_glrlm_LongRunLowGrayLevelEmphasis \| \| SAG_wavelet.LLL_firstorder_MeanAbsoluteDeviation \| \| SAG_wavelet.LLL_firstorder_Skewness \| \| SAG_wavelet.LLL_glszm_SizeZoneNonUniformity \| \| SAG_square_firstorder_RobustMeanAbsoluteDeviation \| \| SAG_logarithm_glszm_GrayLevelNonUniformityNormalized \| \| SAG_logarithm_ngtdm_Busyness \| \| SAG_logarithm_ngtdm_Contrast \| |
| TRA feature group  (n=24) | \| TRA_original_shape_MajorAxisLength \| \| --- \| \| TRA_original_shape_MinorAxisLength \| \| TRA_original_glszm_LargeAreaEmphasis \| \| TRA_original_glszm_ZoneVariance \| \| TRA_wavelet.LLH_glrlm_ShortRunEmphasis \| \| TRA_wavelet.LLH_gldm_DependenceNonUniformityNormalized \| \| TRA_wavelet.LHH_glrlm_LongRunLowGrayLevelEmphasis \| \| TRA_wavelet.HLL_firstorder_Uniformity \| \| TRA_wavelet.HLL_glszm_GrayLevelNonUniformityNormalized \| \| TRA_wavelet.HLL_glszm_ZoneEntropy \| \| TRA_wavelet.HLL_ngtdm_Coarseness \| \| TRA_wavelet.HHL_gldm_SmallDependenceHighGrayLevelEmphasis \| \| TRA_wavelet.LLL_glszm_LargeAreaEmphasis \| \| TRA_wavelet.LLL_glszm_ZoneVariance \| \| TRA_square_glszm_LargeAreaEmphasis \| \| TRA_square_glszm_LargeAreaHighGrayLevelEmphasis \| \| TRA_square_glszm_ZoneVariance \| \| TRA_squareroot_firstorder_Skewness \| \| TRA_logarithm_firstorder_Kurtosis \| \| TRA_logarithm_firstorder_RootMeanSquared \| \| TRA_logarithm_glszm_LargeAreaEmphasis \| \| TRA_logarithm_glszm_ZoneVariance \| \| TRA_exponential_glszm_LargeAreaEmphasis \| \| TRA_exponential_glszm_LargeAreaHighGrayLevelEmphasis \| |
| SAG+TRA feature group  (n=57) | \| TRA_original_shape_LeastAxisLength \| \| --- \| \| TRA_original_shape_MinorAxisLength \| \| TRA_original_glszm_LargeAreaEmphasis \| \| TRA_original_glszm_ZoneVariance \| \| TRA_wavelet.LLH_firstorder_Kurtosis \| \| TRA_wavelet.LLH_glszm_SmallAreaLowGrayLevelEmphasis \| \| TRA_wavelet.LLH_gldm_DependenceNonUniformityNormalized \| \| TRA_wavelet.LHL_glrlm_LongRunEmphasis \| \| TRA_wavelet.LHL_gldm_DependenceNonUniformityNormalized \| \| TRA_wavelet.HLL_glszm_GrayLevelNonUniformityNormalized \| \| TRA_wavelet.HLL_ngtdm_Coarseness \| \| TRA_wavelet.HHL_glszm_SizeZoneNonUniformityNormalized \| \| TRA_wavelet.HHL_gldm_SmallDependenceHighGrayLevelEmphasis \| \| TRA_wavelet.HHH_firstorder_Minimum \| \| TRA_wavelet.HHH_glszm_SmallAreaLowGrayLevelEmphasis \| \| TRA_wavelet.LLL_firstorder_Skewness \| \| TRA_wavelet.LLL_glszm_LargeAreaEmphasis \| \| TRA_wavelet.LLL_glszm_ZoneVariance \| \| TRA_square_glszm_LargeAreaEmphasis \| \| TRA_square_glszm_LargeAreaHighGrayLevelEmphasis \| \| TRA_square_glszm_ZoneVariance \| \| TRA_squareroot_firstorder_Skewness \| \| TRA_squareroot_glszm_LargeAreaEmphasis \| \| TRA_logarithm_firstorder_Kurtosis \| \| TRA_logarithm_firstorder_RootMeanSquared \| \| TRA_logarithm_glszm_LargeAreaEmphasis \| \| TRA_logarithm_glszm_ZoneVariance \| \| TRA_exponential_glszm_LargeAreaEmphasis \| \| TRA_exponential_glszm_LargeAreaHighGrayLevelEmphasis \| \| SAG_original_shape_Flatness \| \| SAG_original_shape_MinorAxisLength \| \| SAG_original_firstorder_RobustMeanAbsoluteDeviation \| \| SAG_wavelet.LLH_firstorder_Skewness \| \| SAG_wavelet.LLH_glszm_GrayLevelNonUniformityNormalized \| \| SAG_wavelet.LHL_gldm_DependenceNonUniformityNormalized \| \| SAG_wavelet.HLL_firstorder_InterquartileRange \| \| SAG_wavelet.HLL_firstorder_Median \| \| SAG_wavelet.HLL_glrlm_GrayLevelNonUniformityNormalized \| \| SAG_wavelet.HLL_glrlm_LongRunLowGrayLevelEmphasis \| \| SAG_wavelet.HLL_glszm_SizeZoneNonUniformityNormalized \| \| SAG_wavelet.HLL_ngtdm_Contrast \| \| SAG_wavelet.HLH_firstorder_Skewness \| \| SAG_wavelet.HHL_firstorder_Kurtosis \| \| SAG_wavelet.HHL_firstorder_Skewness \| \| SAG_wavelet.HHL_glrlm_LongRunLowGrayLevelEmphasis \| \| SAG_wavelet.HHL_ngtdm_Contrast \| \| SAG_wavelet.LLL_firstorder_MeanAbsoluteDeviation \| \| SAG_wavelet.LLL_firstorder_Skewness \| \| SAG_wavelet.LLL_glszm_SizeZoneNonUniformity \| \| SAG_wavelet.LLL_glszm_ZonePercentage \| \| SAG_square_firstorder_RobustMeanAbsoluteDeviation \| \| SAG_squareroot_firstorder_Energy \| \| SAG_squareroot_firstorder_TotalEnergy \| \| SAG_squareroot_ngtdm_Busyness \| \| SAG_logarithm_ngtdm_Busyness \| \| SAG_logarithm_ngtdm_Contrast \| \| SAG_gradient_glrlm_LongRunHighGrayLevelEmphasis \| |

In the WHO tumor grade prediction task, 24 features were selected for SAG, 6 for TRA, and 20 for the SAG+TRA feature group. In the ATRX prediction task, we selected 3 features in the SAG, 39 in the TRA, and 5 in the SAG+TRA feature group. In the P53 prediction task, we selected 21 features in the SAG, 24 in the TRA, and 57 in the SAG+TRA feature group

SAG, sagittal; TRA, transverse
