# Supplementary material for: Multimodal-based machine learning strategy for accurate and non-invasive prediction of intramedullary glioma grade and mutation status of molecular markers: a retrospective study
Source: BMC Med. 2023 May 29;21:198. doi: 10.1186/s12916-023-02898-4 (PMC10228074; doi:10.1186/s12916-023-02898-4)
Supplement: Supplementary file 9 — Additional file 9. Detailed results of the 5-fold cross-validation for the proposed models using multimodal fusion features in the primary cohort. Acc: accuracy, Sens: sensitivity, Spec: specificity. The above results are expressed using the mean of 5 experiments and the corresponding 95% confidence interval. [file 12916_2023_2898_MOESM9_ESM.docx]

**Additional file 9. Detailed results of the 5-fold cross-validation for the proposed models using multimodal fusion features in the primary cohort**

| Measure | WHO-Mind | ATRX-Mind | P53-Mind |
| --- | --- | --- | --- |
| Acc | 89.64% ± 2.43% | 89.04% ± 0.78% | 83.98% ± 5.41% |
| Sens | 54.00% ± 12.93% | 74.00% ± 5.08% | 44.46% ± 17.27% |
| Spec | 94.02% ± 2.23% | 87.43% ± 1.80% | 93.17% ± 3.90% |
| F_1_ | 63.69% ± 11.17% | 77.13% ± 3.06% | 51.33% ± 19.77% |

Notes: Acc: accuracy, Sens: sensitivity, Spec: specificity. The above results are expressed using the mean of 5 experiments and the corresponding 95% confidence interval.
