# Supplementary material for: Multimodal-based machine learning strategy for accurate and non-invasive prediction of intramedullary glioma grade and mutation status of molecular markers: a retrospective study
Source: BMC Med. 2023 May 29;21:198. doi: 10.1186/s12916-023-02898-4 (PMC10228074; doi:10.1186/s12916-023-02898-4)
Supplement: Supplementary file 10 — Additional file 10. Detailed test results in the external test cohort. Acc: accuracy, Sens: sensitivity, Spec: specificity. [file 12916_2023_2898_MOESM10_ESM.docx]

**Additional file 10. Detailed test results in the external test cohort**

| Measure | WHO-Mind | ATRX-Mind | P53-Mind |
| --- | --- | --- | --- |
| Acc | 88.89% | 88.89% | 71.43% |
| Sens | 33.33% | 62.07% | 55.56% |
| Spec | 100% | 100% | 66.67% |
| F_1_ | 46.15% | 72.00% | 52.63% |

Notes: Acc: accuracy, Sens: sensitivity, Spec: specificity.
